# Plasmon–exciton polaritonic emission lifetime dynamics under strong coupling

**DOI:** 10.1515/nanoph-2025-0129

**Published:** 2025-06-24

**Authors:** Povilas Jurkšaitis, Justina Anulytė, Evita Spalinskaitė, Ernesta Bužavaitė-Vertelienė, Vytautas Žičkus, Dovydas Banevičius, Karolis Kazlauskas, Zigmas Balevičius

**Affiliations:** Plasmonics and Nanophotonics Lab., Department of Laser Technologies, Center for Physical Sciences and Technology, Saulėtekio av. 3, LT-10257 Vilnius, Lithuania; School of Physics and Astronomy, University of Glasgow, Glasgow G12 8QQ, UK; Vilnius University, Faculty of Physics, Institute of Photonics and Nanotechnology, Saulėtekio al. 3, LT-10257 Vilnius, Lithuania

**Keywords:** strong coupling, plasmonics, fluorescence lifetime

## Abstract

In this study, we investigate the contribution of resonant and non-resonant excitation conditions on the polariton decay dynamics of strongly coupled rhodamine 6G (R6G) and surface plasmon polariton (SPP). We showed proof of strong coupling between SPP and R6G exciton from the dispersion relations measured by total internal reflection ellipsometry (TIRE). From these it was determined that the coupling strength reaches *g* ≈ 200 meV. Further fluorescence methods were employed to demonstrate the emission from the lower polariton branch (LP). The fluorescence lifetime and back focal plane imaging techniques were implemented to study radiative polariton decay, for resonant and non-resonant excitation conditions. Fluorescence decay measurements of plasmonic strong coupling regime showed considerably longer (ps) than expected lifetime values (fs). In our case the measured lifetimes cannot be explained without the influence of additional energy level in emission dynamics, such as incoherent transition from exciton reservoir to lower polaritonic branch. The fundamental understanding of coherent energy exchange dynamics has potential importance for development of quantum optical nanodevices, polaritonic lasers, polariton condensation.

## 1 Introduction

Plasmonics play an important role in the development of coherent light quantum nano-emitters for advanced optical sensing and integration in electronic circuits. Surface plasmons are coherent oscillations of free electrons and under appropriate conditions can directly interact with light to form surface bound states known as surface plasmon polaritons (SPP). These modes produce optical field confinement and field enhancement at the metal-ambient interface, making them sensitive to changes of the refractive index near the metal surface. Moreover, the conditions for excitation of plasmonic resonance can be altered by changing the metallic nanostructure geometry (corrugated surface or nanoparticles) and arrangement of it. The alteration of geometry allows to tune the resonance properties and improve the sensitivity to refractive index changes in the dielectric environment, making plasmonic structures attractive for optical sensing [1]. The electric field confinement at volumes below the diffraction limit allows to facilitate coupling between the emitters and SPP in the near field [2], [3]. Such plasmonic enhancement of the local electromagnetic field has already been used to boost the optical processes such as fluorescence [4], [5], lasing [6], [7], Raman scattering [8]. It was experimentally demonstrated that when coupling between plasmon and emitter is achieved the spontaneous emission is modified, due to local changes in photon density of states. This results in enhancement or suppression of spontaneous emission rates corresponding to changes in the lifetime of fluorescent sources [9], [10], [11], [12] and is described by Purcell factor *F*_*P*_ = *τ*_*cav*_/*τ*_*fs*_, where *τ*_*cav*_ and *τ*_*fs*_ are spontaneous emissions rates of emitter coupled to cavity mode and in free space, respectively. Despite the advantages, one of the biggest drawbacks of using plasmonic materials remain to be high losses in metal nanostructures, which must be mitigated. When the interaction between SPPs and emitters becomes stronger and overcomes the damping rates of plasmonic cavity and emitter, strong coupling regime can be achieved [13]. It is described as coherent dipole interaction between an emitter and electromagnetic mode. Classically this can be described as a simple coupled harmonic oscillator, which is able to demonstrate many characteristic features of strong coupling. Additionally, semiclassical and quantum models give insight about phenomena such as vacuum Rabi oscillations, Dicke superradiance, electromagnetically induced transparency, etc. Upon strong coupling plasmonic polariton states are formed with unique features resulting in Rabi oscillations between different states which can exchange energy on the femtosecond timescale [14], [15]. These newly formed plasmon–exciton polaritonic states exhibit modified dispersion relations, inheriting properties from both SPP’s and fluorescent molecules. The dispersive characteristic has been a primary feature of polaritonic quasiparticles. Through the use of Hopfield coefficients [16], it is possible to describe polaritonic states as coherent mixture of light-like and matter-like mode, with varying degree of weight. Thus, strong coupling can be used as a technique to tune physical, optical [17], [18], [19] and chemical properties of the emitter, as well as photonic (plasmonic) mode. While a lot of attention of the current research in plasmonic strong coupling is focused on coherence properties of the emitted light [20], [21], [22] and modified chemical dynamics [23], [24], [25], [26], the decay mechanisms within plasmonic strong coupling regime remain rather unexplored [27]. It has been known that due to different mixing ratios between light-like and matter-like components, the polariton fluorescence lifetime should also exhibit dispersive behaviour. However experimental observations have been complicated, resulting in polariton bottleneck effect, where dispersive decay properties are absent [28]. This polariton bottleneck effect is the consequence of slow internal relaxations from exciton reservoir (uncoupled molecules) to polariton state resulting in prolonged lifetimes [29] when non-resonant excitation is applied. Moreover, direct observation of exciton reservoir can be complicated when employing only spectroscopic measurements [30], thus to gain insight about the transition dynamics, time-resolved experiments need to be carried as well. Experiment involving time-resolved resonant pump-probe technique have already confirmed the existence of coherent and incoherent states within strongly coupled systems [31]. However, widely used fluorescence lifetime measurements for organic dye molecules do not demonstrate direct evidence of the strong coupling through the relaxation times. In this study we investigate polariton dynamics within the strongly coupled Rhodamine 6G and SPP system by spectroscopic and temporal measurements. Here we demonstrate the proof of strong coupling by TIRE and fluorescence methods, where the emission from the lower polariton branch (LP) is observed. We also present the concept for the fluorescence decay mechanism from polaritonic state interacting with exciton reservoir, where we define exciton reservoir as uncoupled molecules remaining after initial excitation of polaritonic state. The interplay between different polariton dynamics mechanisms is observed by gradually transitioning from non-resonant excitation conditions primarily linked with bare exciton, to resonantly pumping upper polariton state. By varying the detuning parameters of strong coupling regime within experimental scheme, we can effectively enhance or suppress coupling due to increased resonant excitation conditions described by $\Omega_{R}\propto \sqrt{N}$ [3].

## 2 Methods

### 2.1 Sample preparation

A sample consisting of thin (20 nm) layer of rhodamine 6G embedded in poly(methyl methacrylate) (PMMA-R6G) formed on 45 nm Ag layer was prepared. A thin 45 nm Ag layer was sputtered on cleaned glass substrate (170 µm cover slip glass (CS)) by magnetron sputtering. To prepare the PMMA-R6G thin films a mixture (2:1) solution of 2 parts PMMA (*c* = 0.1 µM, *c*_*m*_ = 0.0367 g/L) and 1 part of R6G (*c* = 25 mM) dissolved in ethanol was used. The 20 nm PMMA-R6G layer was then formed on the top of the Ag metal layer by spin-coating the mixture at 3,000 rpm for 30 s. Additionally, a CS/PMMA-R6G and CS/Ag/PMMA samples with the same thickness were prepared for the reference measurements (ellipsometry and photoluminescence).

### 2.2 Spectrocopic ellipsometry

**Figure 1:**
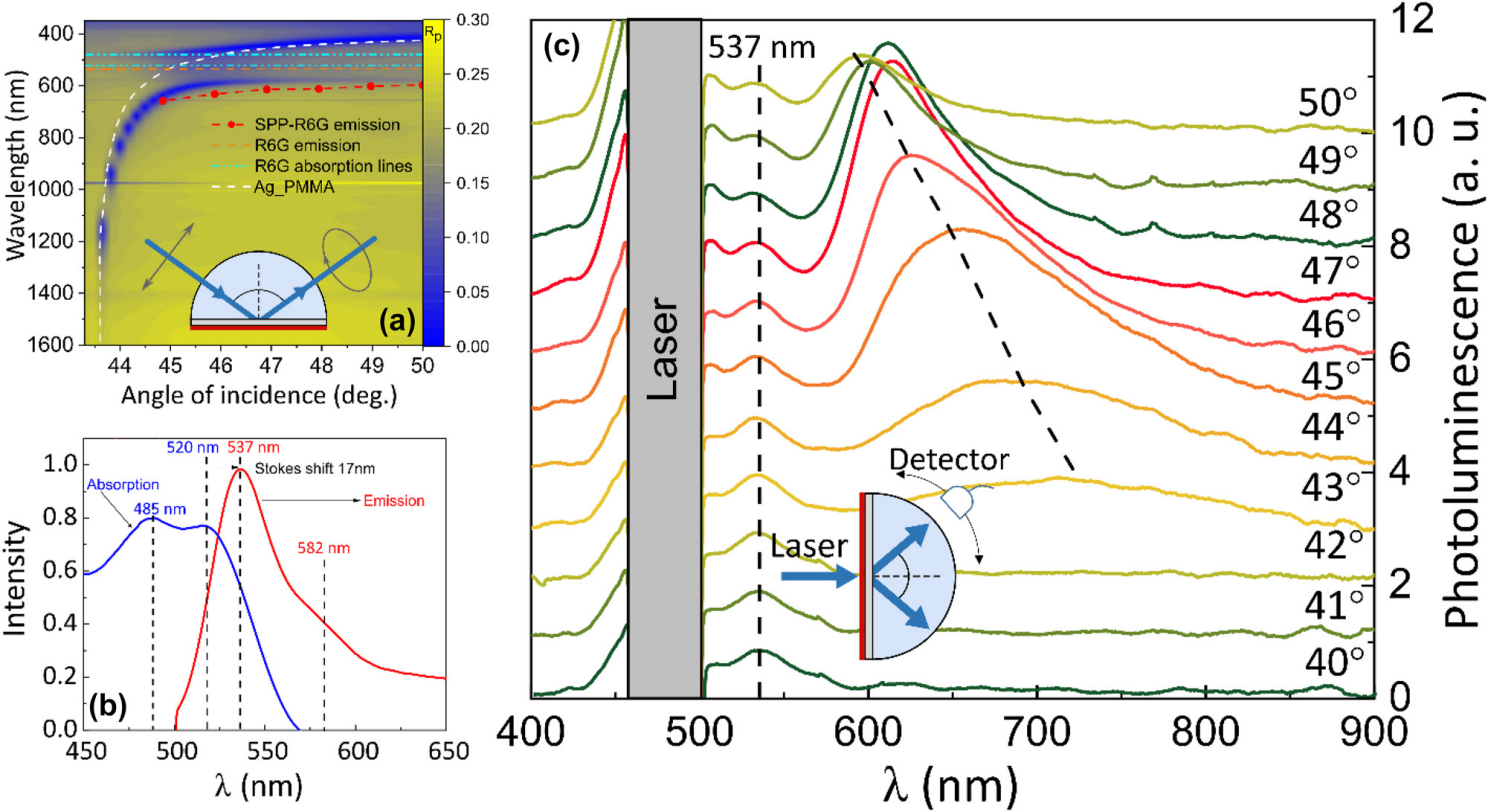
Experimental setup and measured reflection and emission spectrum of strongly coupled SPP and exciton of R6G molecules: (a) total internal reflection ellipsometry measurements of strongly coupled R6G dye and SPP with optical excitation configuration (inset) and emission of SPP-R6G (red symbol curve), single SPP (white dashed line), single R6G absorption (cyan lines) and emission (orange line); (b) typical R6G dye fluorescence absorption and emission spectrum; (c) photoluminescence spectra of strongly coupled SPP-R6G mode at emission angles from 40° to 50°, where the dashed lines are the emission peaks. Schematic representation of photoluminescence experiment setup in shown in the inset.

The dispersion relations of the Ag/PMMA-R6G sample were measured by spectroscopic ellipsometry in total internal reflection geometry. The ellipsometer used was J. A. Woolam RC2 model with two rotating compensators. The light source in RC2 was Xe lamp with spectral range 210–1700 nm, where the optical signal is registered by CCD camera. The sample was attached to BK7 45° angle prism via BK7 refractive index-matching oil. The measurements were conducted in a 43°–50° range angle of incidence (Figure 1(a)). Additionally, the contribution of pure PMMA (*d* = 20 ± 2 nm thickness) layer to the optical response was measured (white dashed curve in Figure 1(a)). The pure PMMA layer shifts the SPP dispersion curve to the longer wavelength, however this red shift had miniscule influence to the coupling strength of SPP and R6G in PMMA layer matrix.

### 2.3 Photoluminescence

The emission of PMMA-R6G and the investigated Ag/PMMA-R6G structure was measured by exciting the samples in reverse Kretschmann configuration (Figure 1(b) and (c)). The excitation was performed from the sample side (normal incidence to the PMMA-R6G surface) and the emission was collected with detector near the glass prism surface (Figure 1(c) inset). The samples were excited with EKSPLA NT tunable laser source, with excitation power of 132 μW at *λ* = 480 nm, where pulse duration was around 4.5 ns. Angle dependent fluorescence detection from samples has been performed using Hamamatsu PMA-12 spectrum analyser, where the probe was placed on a rotating stage.

### 2.4 Back focal plane and fluorescence lifetime measurements

Surface plasmon coupled emission measurements were performed on in-house build microscopy setup. Excitation was performed in Total Internal Reflection Fluorescence (TIRF) configuration with *λ* = 473 nm ps laser source. The substrate side of the samples were placed on Nikon 100X Apo TIRF objective (NA 1.49) connecting it through BK7 index-matching oil. Back focal plane of the microscope objective was sent through 4*f* imaging setup and the light refracted/emitted from the samples was collected on CMOS camera (FLIR Blackfly). Before the 4*f* lens setup, a dichroic filter (FITC) and long pass filter (*λ* = 550 nm) were placed and used to filter out *λ* = 473 nm laser. Additionally, polarizer was inserted in the collection arm (*n* = 1.52) to demonstrate the emission is from polaritonic state.

The lifetimes of fluorescent samples were measured on the same microscopy setup as in BFP measurements in TIRF configuration. The excitation was performed with picosecond diode laser (excitation frequency of 50 MHz at *λ* = 473 nm) with average power of the laser set to 60.5 μW. The samples were excited, and fluorescence was collected from the back side of the sample (substrate). The emitted light is then collected in IDQ single photon detection module where generated electronical signal is registered in PicoQuant MultiHarp150 TCSPC unit.

## 3 Results and discussion

It is well known [32], [33] that there always exists a probability for the non-coherent and coherent transitions to occur in macroscopic quantum systems. Macroscopic quantum coherence phenomenon emerges as the main signature of strongly coupled plasmon–exciton polaritonic states. The different contributions from non-coherent (weak coupling) and coherent (strong coupling) states were previously investigated to study the impact on spatial coherence in frequency domain [34]. Also pump-probe (time domain) reflectance methods were applied to investigate the strongly coupled surface lattice plasmonic modes with J-aggregates and to show the contribution of radiative damping to the optical response [29]. Meanwhile, fluorescence lifetime measurements of strongly coupled polaritonic states together with emission intensity measurements under resonant and non-resonant conditions can reveal the interplay between coherent and non-coherent transition dynamics in strongly coupled polaritonic states.

The investigated structure consisted of thin metal (Ag) film deposited on a glass substrate (CS) with PMMA-R6G layer on top of Ag. The angle dependent reflection and photoluminescence properties of the prepared structure were investigated using three different experimental schemes: total internal reflection ellipsometry (TIRE), angle dependent photoluminescence setup and fluorescence lifetime microscopy setup. Total internal reflection ellipsometry method was performed to retrieve the plasmon–exciton dispersion relation from the investigated sample (CS/Ag/PMMA-R6G) (Figure 1(a)). The measurements were performed using a BK7 glass prism attached to the base of CS connected via refractive index matching liquid, where the excitation and detection is shown in Figure 1(a) inset. First, a CS/Ag structure with 20 nm PMMA layer on top was measured to obtain the surface plasmon polariton dispersion relations (white dashed curve in Figure 1(a)). To obtain the absorption lines of PMMA-R6G ((Figure 1(a) cyan dashed lines)) the *p*-polarization reflection intensity of CS/PMMA-R6G sample was also measured (Figure 1(b) blue curve). Then a CS/Ag/PMMA-R6G sample was measured under the same conditions to evaluate the PMMA-R6G layer contribution to the reflection intensity. From the *p*-polarized reflection dependence on angle of incidence (AOI) and energy (Figure 1(a)) a clear bending of the dispersion lines is observed, where the plasmon–exciton dispersion relations (CS/Ag/PMMA-R6G sample) bend compared to single SPP (CS/Ag/PMMA) and single PMMA-R6G (CS/PMMA-R6G) dispersion curves at the anti-crossing point. This is indicative of strong coupling between SPP and R6G dye exciton [35], [36], [37], [38], [39]. The splitting of the plasmon–exciton dispersion lines in turn allows to evaluate the coupling strength of the hybrid SPP-exciton mode, particularly Rabi splitting (Ω_*R*_) which is proportional to the coupling strength as Ω_*R*_ = 2 g [13]. To determine the hybrid plasmon–exciton mode Rabi gap, the dispersion relations of hybrid and single modes were recalculated to obtain the energy dependence on wavevector. In our case the value of Rabi splitting in *k*-space at the anti-crossing point (*k* = 12.8 µm^−1^) was around 400 meV (*g* = 200 meV). The mode volume of SPP is much smaller than that of purely photonic cavity since the light is confined into the nanoscale volumes. Such plasmonic field intensity is enhanced due to near field and resonant nature of the SPP excitation. On the other hand, the relatively large dipole moment is achieved by high concentration of active media (Rhodamine dye). Thus, it is possible to achieve the vacuum Rabi splitting for so called open cavity (SPP) and emitters without the need of closed cavity metal–insulator–metal what is often used to achieve strong coupling regime [40].

**Figure 2:**
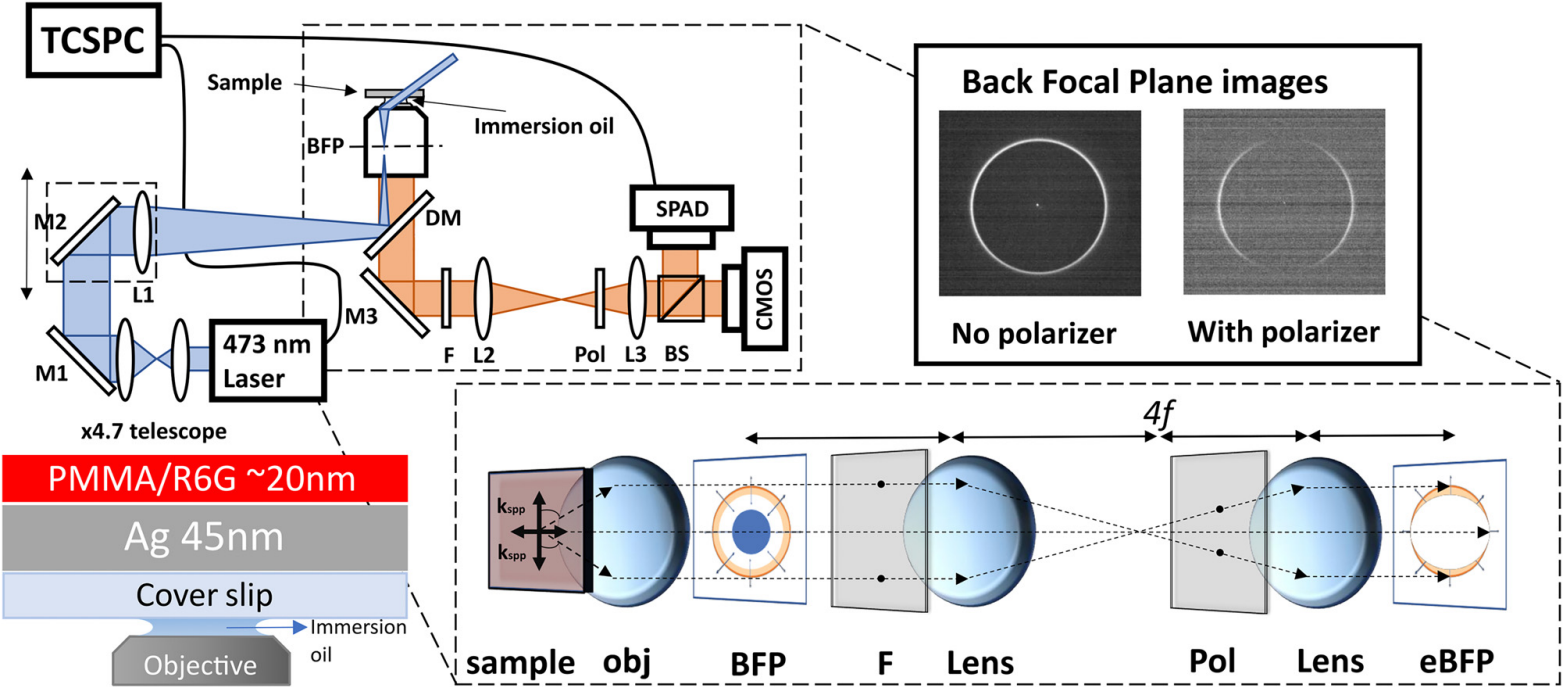
Schematic representation of experimental setup using TIRF excitation of polaritonic modes. The inset shows image formation at back focal plane (BFP) of coupled R6G sample. The investigated CS/Ag/PMMA-R6G sample structure and excitation configuration is shown at the bottom left.

Photoluminescence measurement has been performed to evaluate the emission of bare PMMA-R6G layer on CS (uncoupled sample) and the strongly coupled plasmon–exciton case (CS/Ag/PMMA-R6G sample). To achieve the conditions required for the SPP excitation, the CS/Ag/PMMA-R6G sample was connected to cylindrical BK7 glass prism through index-matching liquid. To have the same excitation conditions as in strongly coupled structure (CS/Ag/PMMA-R6G), the bare PMMA-R6G layer sample was also attached to cylindrical BK7 glass prism. Both samples were excited in reverse Kretschmann configuration (non-resonant condition) using a pulsed 480 nm laser directed normal to the sample surface (air/PMMA-R6G interface) (Figure 1(b) and (c)). The radiation from prism was collected using fiber optic cable mounted on a rotation stage in a range of angles from 40° to 50°. First, the CS/PMMA-R6G sample was measured and the photoluminescence showed no dependence on the emission angle with the main emission peak at 537 nm, indicating fluorescence from bare exciton (transition from excited state to the ground state) (Figure 1(b)). Contrary to the single PMMA-R6G layer, the sample in strong coupling regime (CS/Ag/PMMA-R6G) showed modified emission spectrum under non-resonant excitation conditions. From the photoluminescence spectrum (Figure 1(c)) two peaks can be observed: one angle independent and another angle dependent. A smaller peak with constant intensity that does not depend on the emission angle can be observed at 537 nm. This angle independent peak matches the regular R6G fluorescence, indicating that a fraction of molecular dyes within the systems do not participate in the strong coupling regime. Meanwhile, a peak whose emission shifts from 670 nm (44° AOI) to 600 nm (50° AOI) is observed at the emission angles range 44°–50°. The angle dependent peak corresponds to the emission from the polaritonic state and follows the behaviour of lower polariton branch (Figure 1(a) red symbol curve). Emission from upper polariton state is not observed, however previous studies have reported similar results due to rapid non-radiative decay pathways within strongly coupled SPP-molecular exciton systems [41], [42]. Additionally, it would be challenging to distinguish between the excitation laser and emission from the upper polariton state due to spectral overlap. The normal mode splitting (or Rabi splitting) is proportional to number density $\sqrt{\frac{N}{V}}$ of emissive species (*N* – number of emitters, *V* – volume). Concentration of emitters influence the dipole moment of the system which manifested themselves as the oscillator strength of the strongly coupled mode. From our estimation which was done from the reflectance *p*-polarized TIRE spectra the ratio of integrals between strongly coupled and R6G dye conventional absorptions peaks was 39 % (SPP-R6G)/61 % (R6G) respectively. Influence of the uncoupled states to the dynamics of polariton decay, cannot be described by the spectral measurements alone, thus, other techniques that can provide spectral and temporal information needs to be applied. Thus, leakage radiation microscopy and fluorescent lifetime measurements were performed using total internal reflection fluorescence (TIRF) in 4*f* optical configuration (Figure 2).

**Figure 3:**
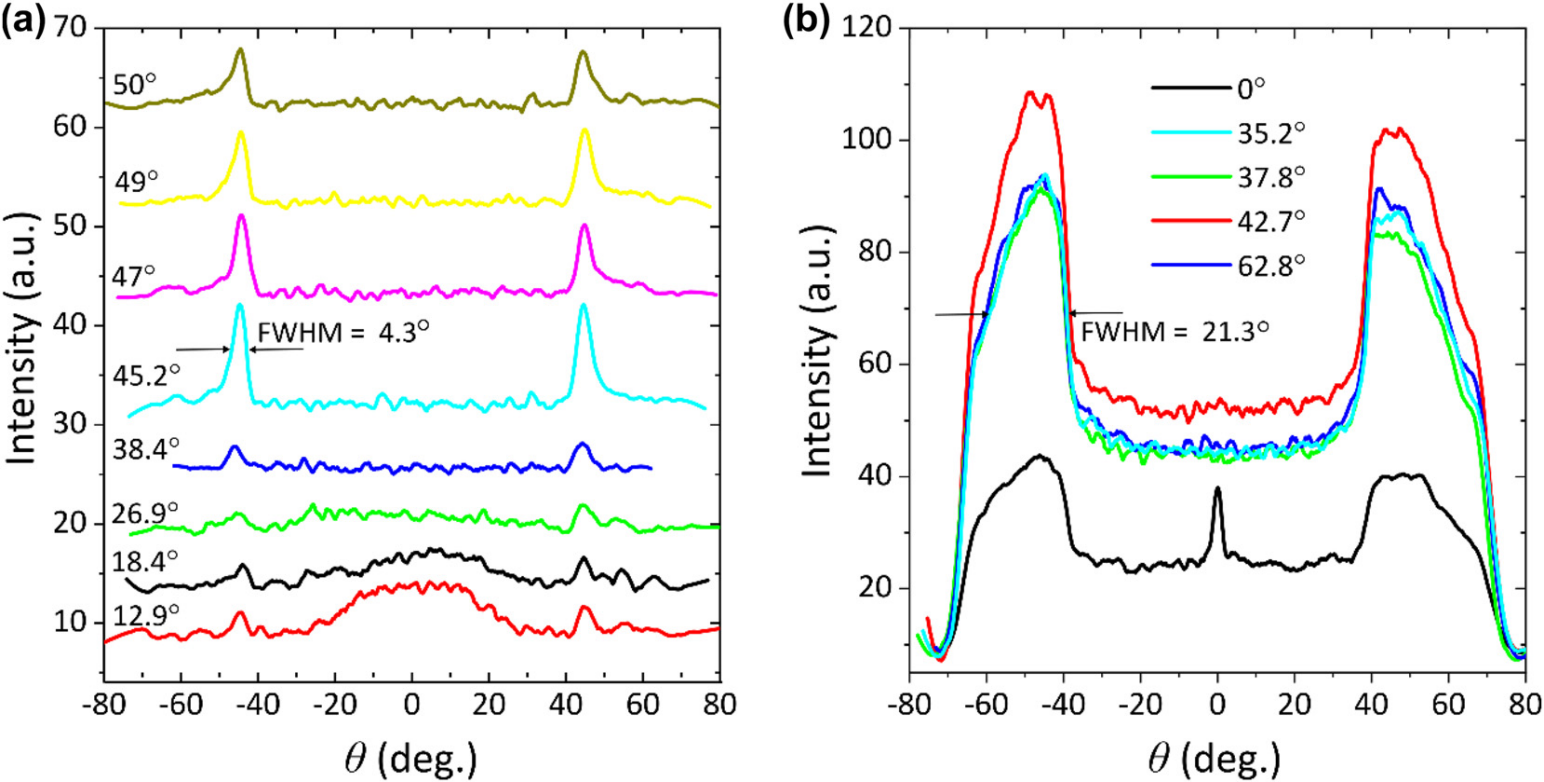
Cross-sectional view in BFP for different laser incident angles: (a) strongly coupled R6G and SPP sample; (b) regular fluorescence emission of R6G, where highest emission intensity is observed to be for 42.7°.

The TIRF microscopy setup with *4f* imaging system used for fluorescence lifetime and leakage microscopy measurements is shown in Figure 2. To perform the fluorescence microscopy measurements of the SPP-exciton emission, the investigated sample was placed on the microscope objective through immersion oil, creating TIRF conditions. Then the sample was excited by 473 nm ps diode laser focusing it on the back focal plane (BFP) of the microscope objective. The narrow angular dispersion light emitted from the sample comes out of the objective and is registered by detector. The image formed at the back focal plane of the objective was reimaged onto CMOS detector (to retrieve BFP image) and single photon avalanche detector (SPAD) (for lifetime measurements) using *4f* imaging system. The resonant plasmon–exciton polariton conditions were achieved by varying the angle of laser incidence to match polaritonic dispersion curve. In order to block the laser light a dichroic mirror and additional long pass filter (FELH0550, Thorlabs) at 550 nm were used, allowing only the emission of wavelengths longer than 550 nm to be registered. Polarizer was inserted between the two lenses in the *4f* imaging system to distinguish between different polarizations. Back focal plane images (Figure 2 top right) of the samples were taken to evaluate the input of the coupling between excitons and SPP to the optical response. Using TIRF setup, the far field emission was investigated for different laser incidence angles ranging from 0° to 62.8°. The horizontal cross section of the BFP images along the centre was taken to analyse the emission of both the single PMMA-R6G layer and the coupled modes (Figure 3). From these cross sections the position of the emission peaks can be determined and related to the emission angles using sin(*θ*) = *NA*/*n* relation.

Figure 3(a) shows cross sectional view of BFP images taken from CS/Ag(45 nm)/PMMA-R6G. The position of the emission peak in strongly coupled case was found to be at 44.1° emission angle. Increasing polariton emission intensity is observed as the incidence angle approaches resonant excitation conditions. In this case, the highest intensity was observed at 45.2° incidence angle. Weak polaritonic emission at 45.2° can also be observed for small angles of incidence (12.9°–38.4°) because of the non-resonant polariton excitation. It is important to mention when small angles of incidence are used in the TIRF setup, part of laser beam still travels at larger angles reaching required conditions for the SPP and exciting the polaritonic state. Comparison of SPP-exciton emission (Figure 3(a)) and regular fluorescence of R6G (Figure 3(b)), shows that the CS/PMMA-R6G(20 nm) sample emits in a wide range of angles with FWHM of 21.3° (Figure 3(b)), whereas fluorescence from the plasmonic structures have emission FWHM of 4.3° (Figure 3(a)). This allows to enhance the collection efficiency of the emitted signal, which is crucial when studying molecular processes [43]. Sharp central peak at 0° AOI in Figure 3(b) (black curve) corresponds to leakage of laser excitation through neutral filters.

**Figure 4:**
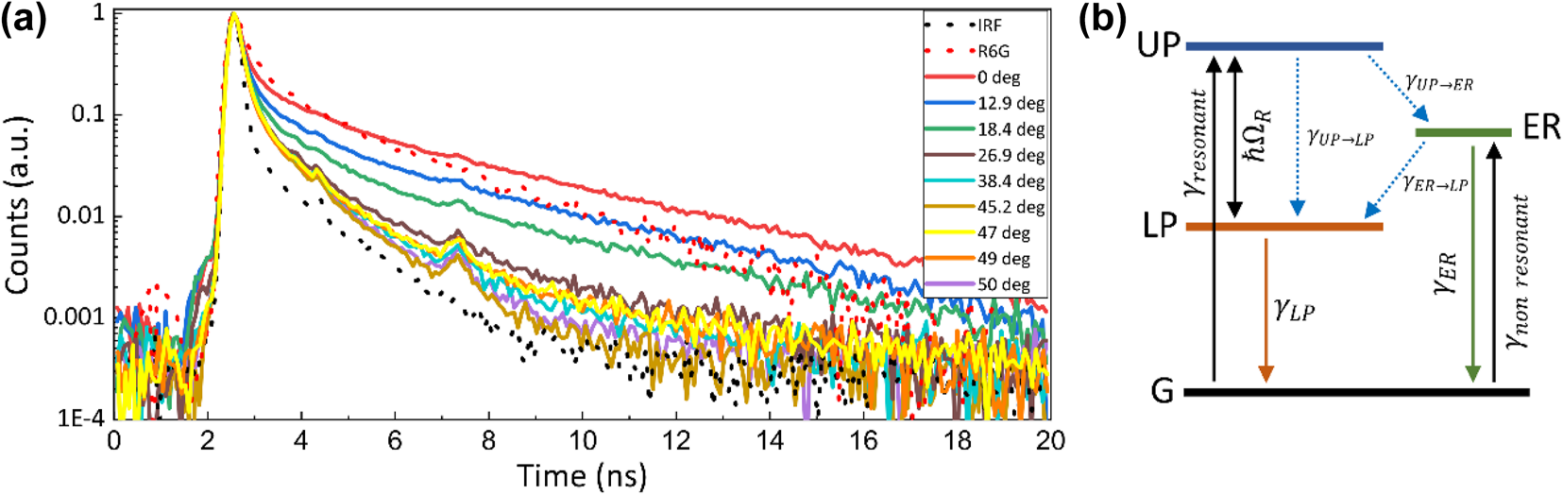
Decay dynamics of strongly coupled SPP-R6G sample: (a) measured fluorescence decay curves of bare R6G-PMMA (red dotted curve) and in strong coupling regime with SPP for different angles of incidence (solid curves). IRF is shown for reference (grey dotted curve); (b) energy level diagram of polariton emission decay pathways from upper polariton state (UP) and exciton reservoir (ER) upon resonant and non-resonant excitation. Internal non-radiative relaxations processes between UP, LP and exciton reservoir are depicted as *γ*_UP→ER_ and *γ*_ER→LP_.

Fluorescence lifetime was examined using time correlated single photon counting (TCSPC) technique (Figure 2). To evaluate the transitions between coherent and non-coherent states, the fluorescence lifetime measurements were performed at different excitation angles. Fluorescence decay was measured for both CS/PMMA-R6G(20 nm) and CS/Ag(45 nm)/PMMA-R6G samples (Figure 4(a)). Measured fluorescence decay data was then fitted with the model function which is the convolution of the instrument response function (IRF) and biexponential function $I\left(t\right)=A_{1}e^{-\frac{t}{\tau_{1}}}+\left(1-A_{1}\right)e^{-\frac{t}{\tau_{2}}}$ , having *R* ^2^ = 0.96. Coefficients *A*_1_ and (1 − *A*_1_) show relative populations of different decay channels within the structure. Pure PMMA-R6G without metal layer sample showed fluorescence lifetime of 0.27 ns. The retrieved value is shorter than typical R6G value of ∼3.8 ns in water solution [44], due to quenching in PMMA matrix. Coupled R6G molecular dye lifetime values were around 0.17–0.15 ns. Retrieved decay profiles can be divided into two groups: for laser incident angles below 18.4° fluorescence decay exhibits longer lifetime, whereas for angles above 18.4° polariton has faster decay. Faster decay times from polariton state, lead to higher emission intensity which can be seen in BFP measurements as shown in (Figure 3(a)). Several decay mechanisms to the ground state are at play upon plasmon-exciton polariton formation (Figure 4(b)). Direct population of the upper polariton (UP) state occurs when resonant excitation is applied followed by rapid (∼50 fs) non-radiative decay to LP state, typical in strongly coupled plasmonic systems [40], [41]. This results in emission from LP branch where decay profile is dependent on the detuning of the cavity mode and no polariton bottleneck is present. In the case of non-resonant excitation direct population of the exciton reservoir was dominant and transition to the LP state occurs through slower non-radiative decay typically in the range of hundreds of picoseconds [28] which then radiatively emits to the ground state. In this case polariton bottleneck effect is present and the dynamics is dominated by the decay rate from the reservoir to the LP state rather than from LP to ground state, resulting in longer decay times. Non-radiative transitions from UP to ER and from ER to LP are depicted as *γ*_UP→ER_ and *γ*_ER→LP_, respectively. In addition, the direct relaxation from the LP to the ground state becomes cumbersome to observe due to limiting IRF which is ∼150 ps.

For further qualitative analysis of contribution the exciton reservoir influence to the lifetimes was simulated by applying rate equation kinetics in which population of polaritonic state was changed from 0 to 0.95. Rate-equation model which describes the population dynamics between excited states and considers possible decay pathways after the exciting the system can be described by the following differential equations:

$$dP/dt=-\left(\gamma_{UP\to LP}+\gamma_{LP\to G}\right)P-\gamma_{UP\to ER}P$$

$$\begin{array}{ll} dE/dt & =\gamma_{UP\to ER}P-\gamma_{ER\to G}E\\ & \;-\left(\gamma_{ER\to LP}+\gamma_{LP\to G}\right)E \end{array}$$

**Figure 5:**
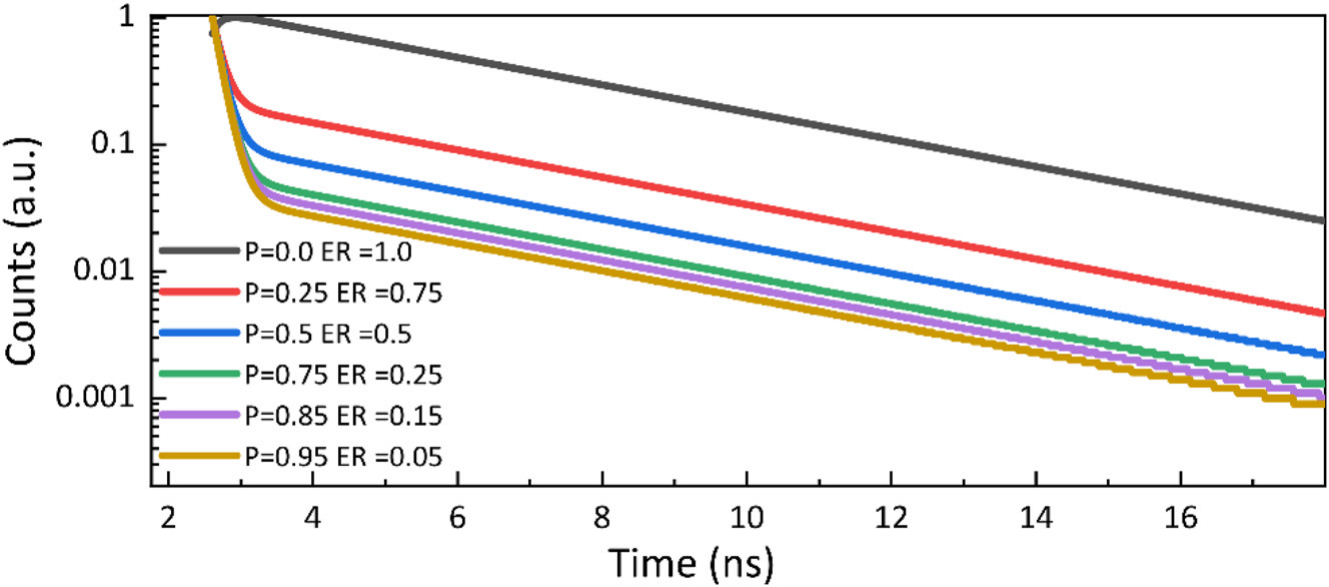
Modelled time dependent emission intensity of the strongly interacting SPP-R6G sample. Values shown below describe the initial populations of the excited UP and ER states.

Solutions to the differential equations, describe decay to the ground state (G) and give total emission:

$$\begin{array}{ll} I\left(t\right) & =\left(\gamma_{UP\to LP}+\gamma_{LP\to G}\right)P\left(t\right)\\ & \;+\left(\gamma_{ER\to LP}+\gamma_{LP\to G}\right)E\left(t\right) \end{array}$$

where, $\left(\gamma_{UP\to LP}+\gamma_{LP\to G}\right)P\left(t\right)$ describes emission from UP–LP–G transition and $\left(\gamma_{ER\to LP}+\gamma_{LP\to G}\right)E\left(t\right)$ emission from ER–LP–G transition, respectively. Due to filtering of the main R6G emission peak, the transition from ER–G is not considered. Modelled results show the emission intensity and its dependence on initial population of UP and ER states. Longer polariton decay lifetime is a result of slow decay through ER–LP–G transition and is known as polariton bottleneck effect. The modelled response in Figure 5 showed lifetimes shortening with decreasing population in ER what follows the tendency of experimentally measured lifetimes in Figure 4(a). Therefore, interactions with exciton reservoir should be considered in order to explain the obtained results.

## 4 Summary

To conclude, several experimental techniques were employed to investigate properties of strongly coupled R6G exciton – plasmon polaritonic state. Spectroscopic ellipsometry in TIRE configuration was used to evaluate the coupling strength indicating clear splitting of the modes, where coupling strength was equal to 200 meV confirming the presence of strong coupling. Photoluminescence measurements have shown two peaks in the emission spectrum. Photoluminescence at lower energy was attributed to the emission of SPP-exciton from lower polaritonic branch, due to angle dependent shift in emission spectrum. While another peak, detected at 537 nm corresponds to uncoupled exciton emission, indicating that some part of excited Rhodamine 6G molecules do not interact strongly with surface plasmon mode. As the angle of incidence increases toward resonant excitation of polariton state, non-resonant excitation of polaritons decreases and the exciton reservoir influence to the dynamics of the system becomes minimal. This implies that typical description of plasmonic strong coupling dynamics characterized by coupled oscillator model do not capture the full picture and additional decay mechanism need to be taken into account. Fluorescence decay measurements showed dependence on excitation conditions with lifetime values in the range of hundreds of picoseconds. This is considerably longer than the expected lifetime values of the order of femtosecond for plasmonic strong coupling regime. In our case the measured lifetimes cannot be explained without the influence of additional energy level in emission dynamics such as incoherent transition from exciton reservoir to lower polaritonic branch. The fundamental understanding of coherent energy exchange dynamics would have potential impact to development of quantum optical nanodevices, polaritonic lasers, polariton condensation.
